# Chemotherapy-Induced Right Ventricular Dysfunction

**DOI:** 10.3390/medicina62091619

**Published:** 2026-08-22

**Authors:** Camil-Horia-Eusebiu Crișan, Anca-Daniela Fărcaș, Andrada-Viorica Pârvu, Mirela-Anca Stoia, Florin-Petru Anton, Angela Cozma

**Affiliations:** 1 4th Department of Internal Medicine, “Iuliu Hațieganu” University of Medicine and Pharmacy, 400012 Cluj-Napoca, Romania; crisancamil@gmail.com (C.-H.-E.C.); mirelastoia@yahoo.com (M.-A.S.); florinantonfr@yahoo.com (F.-P.A.); angelacozma@yahoo.com (A.C.); 2 Department of Internal Medicine, Satu Mare Emergency County Hospital, 440192 Satu Mare, Romania; 3 Department of Cardiology, Cluj Emergency County Hospital, 400006 Cluj-Napoca, Romania; 4 10th Department of Oncology, “Iuliu Hațieganu” University of Medicine and Pharmacy, 400012 Cluj-Napoca, Romania; parvuandrada@hotmail.com; 5 Department of Hematology, “Prof. Dr. Ion Chiricuță” Oncology Institute Cluj-Napoca, 400015 Cluj-Napoca, Romania; 6 Department of Internal Medicine, “Căile Ferrite” Hospital Cluj-Napoca, 400015 Cluj-Napoca, Romania

**Keywords:** chemotherapy, right ventricular failure, right ventricular dysfunction, heart failure

## Abstract

The right ventricle has been considered by many clinicians to be the “forgotten chamber” of the heart. In this narrative review of the literature, we aim to examine chemotherapy-induced right ventricular failure. We will discuss the clinical significance of the subject, the effects of chemotherapy on the heart, and why it is crucial to take a closer look at the right ventricle. We will present key aspects of the anatomy and physiology of the right ventricle and how it differs from the left ventricle. Furthermore, this review explains the mechanisms behind chemotherapy-induced right ventricular dysfunction, strategies for assessing the right ventricle, recent advancements in cardiac imaging, available treatment options, and future directions in therapeutic management.

## 1. Introduction

The field of oncology has always been at the forefront of medical therapeutic advances. New molecules are continuously being researched, and the therapeutic guidelines are constantly evolving and improving with the advent of novel treatment plans. The guidelines that we were relying on not more than 10 years ago are nothing more than history today. That is how fast new molecules and treatment regimens are being discovered, and the benefits are clear: higher survival rates, longer disease-free periods and an overall reduction in morbidity and mortality induced by cancer. Since the 1970s, cancer treatment has greatly improved, and it is now widely accepted that the most effective management of systemic cancer is treatment with standard or high-dose chemotherapy. It has been estimated that 50% of all cancer patients will be treated with some form of chemotherapeutic agent [1]. Due to the increase in survival rates in recent years, there has also been an increase in complications related to long-term exposure to chemotherapy. More patients are succumbing not to the cancer itself but to complications stemming from prolonged treatment regimens or cumulative dose-dependent toxicities, particularly those affecting the heart. The cardiotoxic effects of anthracyclines have been extensively studied, and other chemotherapeutic agents, notably immune checkpoint inhibitors and proteasome inhibitors, have also been implicated in heart failure. A high prevalence of left ventricular dysfunction in long-term survivors of malignancy has been reported [2,3]. Nevertheless, a review of the literature revealed that the right ventricle has been the “ignored sibling” throughout these studies.

### 1.1. The Background of Chemotherapy and Its Effects on the Heart

Chemotherapy is one of the main modalities of treatment in cancer. Although it is not new, the advent of new chemotherapeutic agents and the ability to combine them with existing and new biological agents has significantly enhanced survival rates for solid and hematologic malignancies. The effectiveness of these newer agents in prolonging life often comes with the expense of toxicities that were not seen with the older agents, possibly due to lower life expectancies. While toxicities can affect various organ systems, one of the most critical impacts is their effect on the myocardium; it is well recognized that certain chemotherapeutic agents or their metabolites are cardiotoxic [3].

One of the first widely recognized chemotherapeutic agents linked to heart failure is the anthracycline class. Anthracyclines are antitumor antibiotics used to treat a wide range of malignancies, being particularly effective against hematologic malignancies and breast cancer. The pivotal retrospective analysis that established that anthracycline-induced congestive heart failure was dose-dependent was published by Dr. Von Hoff and colleagues in 1979 [4,5]. Since then, anthracyclines have been associated with congestive heart failure (CHF) and left ventricular (LV) dysfunction. Newer agents, like trastuzumab, a monoclonal antibody, or proteasome inhibitors demonstrated an exacerbation of heart failure in patients with pre-existing heart disease. This could lead to the discontinuation of chemotherapy and an increase in morbidity and mortality for these cancer patients. It is thus becoming increasingly more important to find ways to prevent these toxicities from occurring [6,7].

### 1.2. Importance of Studying Right Ventricular Dysfunction

The mechanisms of how right ventricular failure can induce left ventricular failure have been documented, but most foundational studies focused on patients with congenital heart disease or primary pulmonary disease. Chung et al. provided an excellent look at the Bernheim syndrome and the evolution of our understanding of the reverse effect where the right ventricle obstructs the left [8]. Motoji et al. conducted a pivotal study on 96 patients with pulmonary arterial hypertension (PAH). Using speckle-tracking and transmitral flow velocity, they demonstrated a direct, significant correlation between impaired RV systolic function and LV under-filling. Their findings confirmed that bi-ventricular interdependence profoundly affects long-term survival in PAH patients [9]. To prove that this phenomenon extends beyond pulmonary disease, Paneni et al. studied the right-to-left ventricular interaction in patients undergoing dialysis. They found that RV dysfunction induced by an arterio-venous fistula (AVF) directly correlated with an impairment of left ventricular contraction and relaxation, confirming that right-sided volume overload triggers left-sided dysfunction, further proving what Walker and Buttrick had already noted about the reverse Bernheim effect and ventricular interdependence [10,11]. A recent state-of-the-art review by Petit and Vieillard-Baron broke down how ventricular interdependence manifests in critically ill patients. They explain that the shared interventricular septum and the acutely inextensible pericardium result in the abnormal filling of the left ventricle when the right ventricle is overloaded, particularly during positive pressure ventilation or acute pulmonary hypertension [12].

Despite this, the RV has often been overlooked in broader studies of acquired heart disease. Globally, few publications focus on this area, and little is known about RV dysfunction in acquired heart disease. Compared to the left ventricle, the right ventricle has a complex geometry that makes it difficult to assess using suboptimal echocardiographic windows. These imaging challenges have led to an underestimation of the prognostic importance of RV dysfunction—an underestimation further reinforced by a relative lack of standardized ultrasound parameters for the RV compared to the LV [13]. This lack of evidence translates to an absence of specific therapeutic guidelines for improving outcomes in patients with RV dysfunction secondary to acquired heart disease. Exacerbating this issue is the current lack of definitively proven cardioprotective measures tailored to prevent chemotherapy-induced right heart failure [3].

## 2. Methods

### 2.1. Literature Search Strategy

To provide a comprehensive overview of this underrepresented topic, we conducted a narrative review of the existing literature regarding chemotherapy-induced right ventricular (RV) dysfunction. A structured literature search was performed utilizing electronic databases, including PubMed/MEDLINE, Scopus and EMBASE. The search encompassed articles published from the inception of the databases up to June 2026.

The primary search strategy employed a combination of the following Medical Subject Headings (MeSH) and free-text keywords: (“right ventricular dysfunction” OR “cardio-oncology” OR “anthracyclines” OR “immune checkpoint inhibitors” OR “targeted therapies”). Boolean operators (AND, OR) were utilized to combine search terms and optimize the retrieval of the relevant literature. The reference lists of the identified articles and relevant guidelines were also manually cross-referenced to ensure no pivotal studies were omitted.

### 2.2. Inclusion and Exclusion Criteria

To maintain the clinical relevance and focus of this review, specific inclusion and exclusion criteria were applied. The inclusion criteria were: 1. peer-reviewed original research articles, meta-analyses, systematic reviews, and clinical trials; 2. studies specifically investigating the effects of chemotherapeutic agents on right ventricular structure, function, or bi-ventricular interactions; 3. studies involving adult human subjects; and 4. articles published in English.

Exclusion criteria encompassed purely animal model studies, pediatric populations, single case reports without broader clinical relevance, non-English-language publications, and abstracts presented at conferences without full-text availability.

## 3. Differences Between Right and Left Ventricular Dysfunction

The variation in the disease processes of each ventricle can be conceptualized by comparing right and left heart failure. While increased afterload and pulmonary disease predominantly affect the right ventricle, most left ventricular diseases impair function due to a primary loss of contractile elements. Right ventricular chemotherapy-induced dysfunction involves both damage to the contractile elements and damage to the tricuspid valve, resulting in regurgitation. The pharmacokinetic factors determining how these cytotoxic drugs accumulate differentially in either ventricle remain poorly understood. Right ventricular function is more dependent upon preload than the left ventricle. Any increases in the afterload of the right ventricle will cause dysfunction. This is important because the afterload of the heart is increased in most patients due to the various cytotoxic drugs used, and any ensuing RV disease often precipitates subsequent left ventricular dysfunction [14,15].

The right and left ventricles are anatomically and functionally distinct. The right ventricle is a thin-walled chamber that is intricately related to the left ventricle. It is a volume pump that propels the deoxygenated blood it receives from the right atrium into the lungs via the low-resistance pulmonary circulation. In contrast, the left ventricle has a much thicker wall and serves as the primary pressure pump of the heart, driving oxygenated blood from the left atrium into the high-resistance systemic circulation via the aorta. The function of the left ventricle is more easily quantifiable due to its straightforward concentric geometry and has historically been considered the most important cardiac chamber in relation to the body.

### 3.1. The Anatomy and Physiology of the Right Ventricle

The right ventricle is the most anterior chamber of the heart. It lies inferior and to the left of the right atrium, almost directly opposite the left ventricle, to which it connects functionally and shares a septum. The RV is roughly triangular in shape; in cross-section, it forms a crescent. The inner surfaces contain prominent, springy, band-like ridges of muscle called trabeculae carneae. On the anterior wall and the septum, there are also several small, conical muscles called papillary muscles. The muscle ridges and papillary muscles support the myocardium and serve to anchor the delicate thread-like chords (chordae tendinae) that attach to the tricuspid valve [16].

While the right and left ventricles pump the exact same volume of blood per minute, they operate under entirely different physical constraints. Because the systemic circuit has high resistance, the left ventricle evolved as a thick, muscular cylinder built for brute force. The pulmonary circuit, however, is a low-pressure, low-resistance system. Therefore, the right ventricle does not require dense muscle; instead, it is a thin-walled, highly compliant, crescent-shaped pouch that wraps around the left ventricle. Unlike the concentric, twisting contraction of the left ventricle, the right ventricle pumps using a distinct three-stage mechanism:

1. Longitudinal shortening: The primary pumping action comes from the RV shortening from its base toward its apex.
2. Free wall movement: The thin outer wall of the RV collapses inward toward the central interventricular septum.
3. Septal traction: As the left ventricle contracts, it mechanically pulls the right ventricle along with it, actively aiding in RV ejection.

The right ventricle’s unique anatomy makes it highly specialized in handling massive increases in blood volume (venous return) but leaves it uniquely vulnerable to sudden increases in pulmonary pressure (afterload) [10,17,18].

### 3.2. Mechanisms of Right Ventricular Dysfunction After Chemotherapy

While cardio-oncology has historically focused almost exclusively on the LV, the RV is far from immune to the toxic effects of chemotherapy. In fact, modern strain echocardiography reveals that subclinical RV damage is frequent and often underdiagnosed and significantly limits a patient’s exercise capacity and survival. A pooled cumulative DOX-equivalent dose of 231.8 ± 4.7 mg/m^2^ has been shown to exert significant toxic effects on the right ventricle [19].

**Figure 1:**
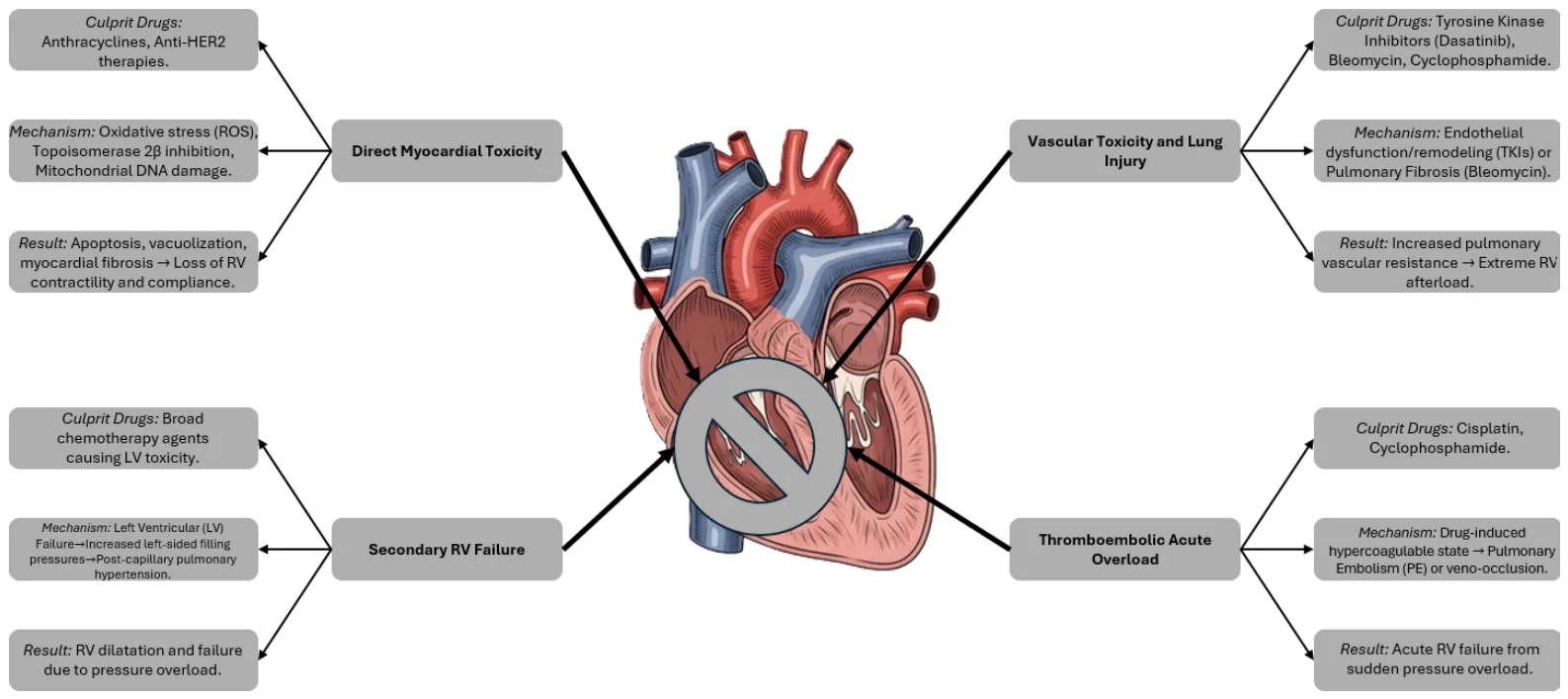
The four distinct pathophysiological mechanisms leading to chemotherapy-induced right ventricular dysfunction: direct myocardial toxicity, secondary failure via left ventricular dysfunction, direct pulmonary vascular/lung toxicity, and acute thromboembolic overload.

There are four distinct pathophysiological mechanisms that can lead to chemotherapy-induced right ventricular failure (as summarized in Figure 1):

#### 3.2.1. Direct Myocardial Toxicity

Until recently, RV failure has been considered extremely rare in chemotherapy patients, largely because it has been overlooked. We now know that certain agents like anthracyclines or anti-HER2 therapies can directly damage the myocardium [20]. Using these agents in combination has an even more devastating effect on the myocardium [21,22]. The thinner wall structure of the RV may render it more susceptible to certain forms of chemotherapy-induced injury, although the exact molecular mechanisms remain incompletely understood [23]. Cellular damage can be induced by oxidative stress (anthracyclines generate massive amounts of reactive oxygen species that overwhelm myocyte antioxidant defenses), direct enzyme inhibition (binding to topoisomerase 2β, leading to lethal DNA double-strand breaks in the myocardium), and the induction of mitochondrial dysfunction (chemotherapy severely damages mitochondrial DNA and disrupts the production of ATP). Each of these mechanisms can lead to apoptosis, vacuolization, and, in the end, replacement with rigid fibrotic tissue. Consequently, the RV loses its compliance and its intrinsic ability to contract [24].

#### 3.2.2. Secondary RV Failure

Secondary RV failure presents a similar mechanism to how global heart failure occurs as it is secondary to LV failure. Chemotherapy directly damages the LV, inducing systolic or diastolic dysfunction. The failing LV cannot pump blood forward efficiently, causing blood to back up into the left atrium, increasing left-sided filling pressures. This elevated pressure is transmitted backwards into the pulmonary vasculature, creating post-capillary pulmonary hypertension. Because the RV is extremely sensitive to any increases in afterload, being forced to pump against this new high-pressure gradient causes it to initially dilate in a struggle to maintain output, before ultimately failing [25,26].

#### 3.2.3. Direct Pulmonary Vascular Toxicity

Certain chemotherapeutic agents do not primarily target the heart muscle; rather, they damage the blood vessels within the lungs or induce pulmonary parenchymal scarring. Tyrosine kinase inhibitors (TKIs), such as Dasatinib, are well-established causes of direct endothelial dysfunction and remodeling in the pulmonary arteries. This drastically increases pulmonary vascular resistance, forcing the right ventricle to pump against an extreme afterload and leading to rapid right heart failure [27,28,29]. Conversely, agents like bleomycin or cyclophosphamide can cause severe scarring of the lung tissue, leading to pulmonary fibrosis, which in turn increases vascular resistance and places severe strain on the right ventricle [20].

#### 3.2.4. Thromboembolic Acute Overload

It is a well-known phenomenon that malignancy creates a prothrombotic state, and the majority of prominent chemotherapies—such as cisplatin and cyclophosphamide—further exacerbate this hypercoagulable state. This state frequently leads to venous thromboembolism, including pulmonary embolism (PE), which causes an acute and sudden pressure overload and subsequent RV failure. Pulmonary veno-occlusive disease can also occur with certain regimens, dramatically increasing vascular resistance [20,30].

To better distinguish the effects of conventional chemotherapy from targeted therapies and to separate direct myocardial injury from secondary dysfunction (such as pulmonary vascular injury and thromboembolism), we summarize the primary drug classes, their mechanisms, and key clinical findings regarding RV measurements in Table 1.

### 3.3. Assessing the Right Ventricle

Even though there are a variety of means to image the right ventricle, diagnostic suspicion is often not raised until the patient has developed significant symptoms. Most investigations of the right ventricle involve assessment while investigating left ventricular disease and comparison with age-matched normal subjects. Due to its anatomical position, the RV is notoriously difficult to assess, sometimes being dubbed the “forgotten chamber”. Echocardiography is the standard for clinical evaluation, though historically, there were fewer standardized parameters for assessing RV function compared to the LV. Parameters like Tricuspid Annular Plane Systolic Excursion (TAPSE), S’ velocity and RV fractional area change (RV FAC) are standard practice. Recently, the 2025 American Society of Echocardiography (ASE) Guidelines for the Assessment of the Right Heart revolutionized these metrics by moving away from binary (normal/abnormal) thresholds toward a severity-graded model to enhance prognostic accuracy. While the baseline cut-offs for normal function remain consistent, dysfunction is now stratified into mild, moderate, and severe categories:

- TAPSE (Normal > 17 mm): Mild, 17–13 mm; moderate, 13–10 mm; severe, <10 mm.
- RV FAC (Normal > 35%): Mild, 35–29%; moderate, 29–22%; severe, <22%.
- S′ velocity (Normal > 9.5 cm/s): Mild, 9.5–7.2 cm/s; moderate, 7.2–5.0 cm/s; severe, <5.0 cm/s.

Furthermore, the 2025 ASE guidelines place a renewed emphasis on RV free wall longitudinal strain (RV FWLS) and RV global longitudinal strain (RV GLS) with severity grading and advocate for 3D RA and RV volumes indexed to body surface area when available, as 3D imaging reduces geometric assumptions. Strain values more negative than −20% to −25% for both RV FWLS and RV GLS are generally considered normal [32].

Employing ultrasound-enhancing agents is also becoming increasingly popular to improve endocardial border visualization [33,34].

When echocardiography is insufficient, more advanced modalities can be used. Cardiac MRI is currently the gold standard for right ventricular assessment, granting us a highly accurate look at the anatomy, volume, and contractility in real time, while right heart catheterization remains the gold standard for direct hemodynamic measurements. Unfortunately, these advanced techniques cannot currently be employed on a routine, large-scale basis for all oncology patients.

A JACC State-of-the-Art Review recommends that baseline and surveillance imaging for patients receiving cardiotoxic agents (such as anthracyclines, HER2-targeted therapies, proteasome inhibitors, MEK inhibitors, and VEGF inhibitors) should routinely include both LV and RV systolic and diastolic function assessment, supplemented by GLS [35,36].

## 4. Consequences of Right Ventricular Dysfunction

### 4.1. Pathophysiological Changes in Right Ventricular Function

The pathophysiological consequences of right ventricular global systolic dysfunction include increases in the duration and magnitude of paradoxical septal motion and significant decreases in left ventricular filling, ultimately leading to decreased cardiac output. In the absence of primary or severe LV abnormality, the systolic dysfunction of the RV is the main cause of a decrease in functional class, exercise performance, and poor outcome in patients with heart failure. RV diastolic dysfunction is technically more difficult to study than systolic dysfunction, but it appears to be highly prevalent in conditions such as valvular disease, ischemic heart disease, and cardiomyopathy. Its importance in the development of RV hypertrophy and RV failure has spurred increased interest in recent years. The most common echocardiographic manifestation of RV diastolic dysfunction is a reduction in early diastolic filling. This is particularly evident in disease states such as pulmonary hypertension, but it is also observed in patients with no obvious structural cardiac abnormalities where the only physiological derangement is chronic hypoxia. Kanagala et al. proved that right ventricular dysfunction is independently associated with an increased risk of death or hospitalization for heart failure, establishing right ventricular dysfunction as an independent predictor of adverse clinical outcomes [37,38].

The RV is unique among cardiac chambers in its strict reliance on physiological interdependence with the LV to maintain cardiac output. Optimal right ventricular function is dependent on maintaining the normal structure and function of the RV myocardium, the pulmonary vasculature, and the left ventricle. The most well-studied cardiotoxic drug is the anthracycline doxorubicin. Anthracyclines disrupt myocardial structure and function by inducing myofibrillar loss, disarray and thinning and by ultimately causing cardiomyocyte death and fibrosis. These pathological changes in the myocardium lead to increased ventricular end-diastolic and -systolic volumes and ultimately a reduction in ejection fraction. Trastuzumab, a monoclonal antibody (mAB) that interferes with the HER2 receptor, produces similar myofibrillar abnormalities and myocyte death [39,40,41].

### 4.2. Clinical Implications and Prognostic Significance

RV function has been increasingly recognized as a crucial determinant of outcomes in patients with heart failure. An increasing body of evidence suggests that RV performance is a major prognostic factor across a diverse range of cardiovascular diseases, and it is also emerging that intrinsic RV dysfunction carries a poor prognosis even in isolation. Chemotherapy has significantly improved the outlook for patients with a variety of malignant disorders, in terms of both longevity and quality of life. However, this improvement has led to an increased awareness of the adverse effects of chemotherapy agents on the cardiovascular system. While most clinical concern has historically focused on left ventricular cardiomyopathy and congestive heart failure linked with left ventricular dysfunction, there is now increasing recognition of the direct and indirect damage inflicted on the right ventricle.

Our search of the literature identified multiple chemotherapy agents implicated in causing RV dysfunction, the most well-recognized being anthracycline agents such as doxorubicin. A systematic review and meta-analysis of fifteen studies conducted by Theetha Kariyanna et al. concluded that chemotherapy with anthracyclines and trastuzumab negatively affects right ventricular function, leading to a significant decline across multiple parameters, including an RV ejection fraction mean difference of 2.70, RV fractional area change mean difference of 3.89, RV GLS mean difference of −1.97, and RV free wall longitudinal strain mean difference of −1.98, paralleling an LV ejection fraction mean difference of 3.88 [25]. Furthermore, an observational study by Planek et al., conducted on 35 lymphoma patients without known baseline heart disease who were treated with doxorubicin, demonstrated the dose-dependent effect of doxorubicin therapy on subclinical RV dysfunction as measured by echocardiography. Based on these findings, the authors strongly recommend that, in addition to LV, routine RV function monitoring serves as a cost-effective tool for the early detection of subclinical cardiac dysfunction during chemotherapy [31].

The broader prognostic weight of the RV is clear: in a study of 625 patients diagnosed with heart failure, 241 patients (38.6%) exhibited RV dysfunction at baseline. The presence of RV dysfunction at baseline was strongly associated with a higher risk of reaching the combined endpoint of all-cause mortality and HF hospitalization over a mean follow-up of 3.3 ± 1.9 years [42].

### 4.3. Therapeutic Measures

Our review of the literature found that at this moment, there are no FDA- or EMA-approved pharmacological therapies that specifically and exclusively target the right ventricular myocardium. In daily clinical practice, we rely almost entirely on therapies designed to treat either left heart failure or pulmonary arterial hypertension (PAH). Therefore, caution is required, as evidence derived from general heart failure or PAH populations cannot be universally applied to chemotherapy-related RV dysfunction without acknowledging the distinct pathophysiological mechanisms at play.

Guideline directed medical therapy should be initiated in every patient exhibiting signs of clinical heart failure [43,44].

While current guideline-directed medical therapies (GDMTs) have been highly effective for the failing left ventricle, their application in primary RV failure requires careful consideration. Evidence drawn from PAH and primary RV failure studies suggests that neurohormonal blockade can yield mixed or even detrimental results. For instance, in certain PAH cohorts, suppressing the RV’s compensatory sympathetic drive with beta-blockers has been shown to worsen hemodynamics and deteriorate cardiac function, as the failing RV relies on sympathetic overdrive to maintain cardiac output [45,46]. Similarly, the routine use of ACE inhibitors, ARBs, and ARNIs is not generally recommended in isolated right heart failure, as codified in the 2022 ESC/ERS Guidelines, due to the risk of hypotension compromising right coronary perfusion [47]. However, it is vital to recognize that chemotherapy-induced dysfunction often involves bi-ventricular toxicity, where these agents remain foundational for LV protection and overall mortality reduction.

Mineralocorticoid receptor antagonists (MRAs) like spironolactone and eplerenone are frequently used in right heart failure. Not only do they help with congestion by having a synergistic effect with loop diuretics to reduce fluid overload, but they also significantly reduce vascular inflammation and improve endothelial function in patients with PAH [48].

Sodium–glucose cotransporter 2 inhibitors (SGLT2is) have proven effectiveness in the treatment of general heart failure. In experimental and PAH models, SGLT2is have been shown to directly alter myocardial metabolism, promoting the utilization of ketone bodies as the pressure-strained RV switches from efficient fatty acid oxidation to inefficient glycolysis [49,50]. While they represent a highly promising crossover therapy, their specific efficacy and safety profile in primary chemotherapy-induced RV failure remains to be established in dedicated clinical trials.

Dexrazoxane has shown efficacy in preventing or reducing cardiotoxicity in adults treated with anthracyclines, although further studies are required to separate its bi-ventricular benefits [51].

Retrospective studies have shown that cancer patients who coincidentally took statins for high cholesterol had significantly lower rates of heart failure. The STOP-CA trial [2023] provided definitive proof for LV protection, showing that patients taking atorvastatin had a nearly 60% reduction in the risk of experiencing a clinically significant decline in LVEF [52]. The obtained results shifted the paradigm, and currently, the 2022 ESC Guidelines on Cardio-Oncology explicitly recommend primary prevention strategies for patients deemed at high or very high risk of cardiotoxicity [3]. However, it is important to emphasize that the STOP-CA trial assessed LV-specific endpoints; its direct applicability to RV-specific outcomes remains an area requiring further investigation.

Patients with anthracycline-induced cardiomyopathy requiring mechanical circulatory support had a significantly increased need for bi-ventricular support due to proportionate RV failure, suggesting that RV dysfunction in this population may be more severe than in other cardiomyopathies [53].

The field of medical therapies that directly target the RV myocardium or RV failure is currently expanding at an increased pace. Different types of medications, such as modulators of metabolism, reactive oxygen species, or inflammation, are currently under development. Furthermore, advanced therapies utilizing microRNA, long non-coding RNA, and cell-based regenerative therapies are showing great promise in preclinical right heart models [54].

## 5. Conclusions

Through this review of the literature, we highlighted the significant impact that chemotherapy exerts on the right ventricle. Right ventricular dysfunction is often underdiagnosed and overlooked, which is highly problematic because subclinical RV dysfunction often occurs much earlier than previously recognized. The old clinical adage of considering the left ventricle as the only one involved in chemotherapy-induced heart failure must be abandoned. The pathological changes that occur in the RV can lead to heart failure which in turn frequently necessitates the cessation of potentially curative chemotherapy treatments, which are vital to achieving disease-free status. A closer look at the right ventricle during regular cardiovascular follow-up allows clinicians to detect early signs of the toxicity of the right ventricle before the left ventricle is irreversibly affected and before decompensated heart failure sets in. This proactive approach enables patients to safely complete their full cancer treatment regimens, ultimately providing them with better chances of surviving their malignancy while preserving their quality of life. Modern echocardiographic strain imaging is becoming increasingly accessible and allows for this crucial early detection when employed properly. Future cardio-oncology guidelines should universally incorporate routine, standardized RV assessments.

## Figures and Tables

**Table 1:** Summary of chemotherapeutic agents and their impact on right ventricular function.

Drug Class and Agents	Representative Study/Reference	Sample Size	RV Measurement(s)	Follow-Up	Main Findings
Anthracyclines and Anti-HER2 (e.g., Doxorubicin, Trastuzumab)	Theetha Kariyanna et al. [25] (Meta-analysis)	644 patients from 15 studies	RVEF, RV FAC, RV GLS, RV FWLS	Less than 12 months since start of chemotherapy	Negatively affects RV function, leading to a significant decline in RVEF (mean diff 2.70), RV FAC (3.89), RV GLS (−1.97), and RV FWLS (−1.98).
Anthracyclines (e.g., Doxorubicin)	Planek et al. [31] (Observational study)	35 lymphoma patients	Echocardiography (subclinical parameters)	6 months	Demonstrated a dose-dependent effect on RV subclinical dysfunction; suggests RV monitoring as a cost-effective tool for early detection.
Anthracyclines (DOX-equivalent)	Faggiano et al. [19] (Systematic Review and Meta-analysis)	1148 patients from 15 studies	Strain echocardiography	N/A	Subclinical RV damage is frequent and underdiagnosed; a pooled cumulative DOX-equivalent dose of 231.8 ± 4.7 mg/m^2^ showed toxic effects on the RV.
Tyrosine Kinase Inhibitors (e.g., Dasatinib)	Özgür Yurttaş et al. [27]; Dumitrescu et al. [28] (Clinical studies/Case series)	N/A	Right heart catheterization/Hemodynamics	N/A	Induces direct endothelial dysfunction and remodeling in pulmonary arteries, leading to severe pre-capillary pulmonary hypertension and secondary right heart failure.
Alkylating Agents/ Glycopeptide Antibiotics (e.g., Cyclophosphamide, Bleomycin)	Tadic et al. [20] (Review)	366 patients from 10 studies	Cor pulmonale/RV strain	N/A	Causes severe scarring of lung tissue, leading to pulmonary fibrosis, which increases pulmonary vascular resistance and RV strain.

## Data Availability

The original contributions presented in this study are included in the article. Further inquiries can be directed to the corresponding author.
